# A comparative study of the attentional blink of facial expression in deaf and hearing children

**DOI:** 10.1177/20416695231182294

**Published:** 2023-07-04

**Authors:** Yu Zhan Yu, Xing Jin, Linxiang Jia

**Affiliations:** 12675 The School of Education Science, Jiangsu Normal University, China; 12675 The School of Education Science, Jiangsu Normal University, China; 12675 The School of Education Science, Jiangsu Normal University, China

**Keywords:** deaf children, attentional blink, facial expression, disgust, fear

## Abstract

The rapid serial visual presentation paradigm was used to investigate differences in the attentional blink between deaf children and hearing children in response to facial expressions of fear and disgust. The results showed that: (1) deaf and hearing children had a higher accuracy rate for T1 with disgustful facial expression than T1 with fear facial expression, (2) There was no significant difference in attentional blink between deaf and hearing children, (3) When T2 appeared at Lag6, the response accuracy of T2 in the disgust T1 condition was lower than that in fear T1 condition. However, no significant difference in T2 at Lag2 was found between the two conditions. The results showed that deaf children and those with hearing were more sensitive to facial expressions of disgust, which captured more attentional resources, and the ability of visual attention of deaf children was not weaker than hearing children.

According to the World Health Organization's World Hearing Report 2021, about 34 million children suffer from hearing loss worldwide. Due to their high degree of hearing impairment, deaf children rely mainly on visual information (Bachara & Phelan, 1980; Hollingsworth et al., 2014; Luo et al., 2003); accordingly, studies focusing on the visual attention of deaf children have become particularly important. However, these results are controversial. Specifically, the view of visual attention deficit holds that the loss of hearing in deaf children will lead to the impairment of other sensory channels (including vision), which is manifested in reduced visual attention ability (e.g., Grafman, 2000; Gu et al., 2019; Wang et al., 2019). On the contrary, the concept of visual attention compensation argues that the loss of hearing in deaf children will lead to the functional compensation of other sensory channels to adapt to the environment, which is reflected in the enhancement of visual attention ability (e.g., Backman & Dixon, 1992; Ferrari et al., 2019).

Visual attention ability can be reflected in individuals’ selective processing of time series stimuli (Zhang & Wang, 2009) and can be evaluated by the degree of the attentional blink (Becker et al., 2021; Broadbent & Broadbent, 1987; Chen & Wang, 2012). Attentional blink refers to the phenomenon in which participants are presented with a series of stimulus streams and are required to report the target (T1) and detection stimulus (T2) after the stimulus stream. By correctly identifying T1, participants can significantly reduce the recognition accuracy of T2 for the next 200 to 500 ms; this phenomenon is caused by limited attention resources. In this regard, individuals put too much attention resources into T1, and the processing ability of T2 is limited (e.g., Olatunji, 2021; Raymond et al., 1992).

So far, only three studies have explored the significance of attentional blink in deaf children; however, the age of the included study participants did not cover the 13 to 15 age group, and inconsistent results were obtained. Dye and Bavelier (2010) found that deaf children (signers who have not received a cochlear implant) aged 7 to 10 presented with significantly more attentional blink than hearing children; but the difference between deaf and hearing people aged 18–40 was not significant. Thakur et al. (2019) found that deaf children (congenital deafness) aged 8.5 to 11.5 had a lower T2 error rate than hearing children. Moreover, Wang, Wang, et al. (2019) found significantly more attentional blink to negative stimuli (negative words) in deaf people(congenital deafness and signers) aged 16 to 20 than hearing people. Therefore, little emphasis has been paid to the significance of attentional blink in deaf children aged 13 to 15. It is widely acknowledged that this age period is a stage of rapid development of the individual prefrontal lobe and gradual improvement of individual cognitive ability (Luciana et al., 2005). Therefore, the age range of participants in this study was limited to 13 to 15 years old.

Nonetheless, Thakur et al. (2019) suggested that the inconsistency in attentional blink results in deaf children might be due to differences in the types of stimulus materials used and the degree of task difficulty in different studies. Previous studies reported that different emotional vocabulary (Anderson & Phelps, 2001) or face materials (Jia et al., 2016; Stein et al., 2010) can affect attentional blink. When T1 is an emotional stimulus, especially a negative emotional stimulus, participants tend to invest too many resources in T1, thus reducing the accuracy of T2 and leading to a larger attentional blink (Mathewson et al., 2008; Mcnair et al., 2017). Moreover, previous studies reported that deaf children are prone to negative emotions (Fellinger et al., 2012; Mukuna & Maizere, 2022). Such individuals show a dominant effect in the cognitive processing of negative emotions, that is, individuals show a priority effect in psychological processing and behavioral response to negative emotions (Kanske et al., 2011; Theeuwes & Van der Stigchel, 2006).

Furthermore, there are differences in the processing of different negative emotions. Fear is an unpleasant emotion generated when individuals are confronted with actual dangerous situations (Marks & Nesse, 1994), while disgust is associated with unpleasant objects (Rozin & Fallon, 1987). In addition to the conceptual level, the differences between fear and disgust are as follows: (1) at the physiological level, disgust tends to stimulate parasympathetic activity, which reduces heart rate, blood pressure, and respiration (Ekman et al., 1983) while fear tends to stimulate sympathetic activity, which accelerates heart rate, blood pressure, and respiration (Lang et al., 1993). (2) In terms of magnetoencephalography, disgust activates the levator labial muscle, but fear does not (Yartz & Hawk, 2002). (3) In terms of brain imaging, fear expression generally activated the amygdala, anterior cingulate gyrus, and prefrontal cortex; in contrast, disgust expression recognition was associated with the insula and basal ganglia region (Lindquist et al., 2012). Other studies have found that disgust induced more significant Early Posterior Negativity (EPN) than a scary picture, while disgust induced a larger amplitude of P2, P3, and Late Positivity Potential than fear (Fang et al., 2019; Wheaton et al., 2013).

Interestingly, studies also found that disgust attracted more attention than fear and occupied more attention resources. In a study by Carretié et al. (2011), subjectively rated disgust and fear-evoking images were used to assess valence and arousal; normal participants aged 19 to 30 years performed relatively worse for numerical categorization tasks when presented with disgust-evoking images. Zeng and Zheng (2019) also found that aversive stimuli attracted more attention resources, and normal participants aged 17 to 24 years with high aversive sensitivity showed higher attentional bias to all stimuli, suggesting that it was more difficult to disengage from disgust than from fear. In a study by Cisler et al. (2009) where the rapid serial visual presentation (RSVP) paradigm was used, normal participants aged 18 to 21 years were found to have more difficulty in attentional disengagement from aversive words. This conclusion is supported by subsequent studies (Chapman et al., 2013). The above studies all found that adults’ attentional blink for disgust was significantly larger than that for fear, and adults’ selective attention ability was higher than that of children (Day, 1980; Hou & Pan, 2015; Wang & Fang, 1992), but it is still unknown whether hearing children's attentional blink for disgust is larger than that for fear. In addition, in deaf children, according to the view of visual attention deficit, the above difference may be more significant than that in deaf children (e.g., Grafman, 2000; Gu et al., 2019; Wang, Wang, et al., 2019); According to the view of visual attention compensation, the above difference may be similar in deaf children and hearing children (e.g., Backman & Dixon, 1992; Ferrari et al., 2019).

Furthermore, emotional words have been used to explore the phenomenon of emotional attentional blink in deaf children (Wang, Lu, et al., 2019). Nevertheless, there are many differences between the processing of emotional words and faces. Firstly, compared with emotional facial expressions, the meaning of emotional words cannot be directly perceived and can only be obtained through semantic processing (Wang, Lu, et al., 2019). Moreover, the emotional effect of emotional words is weaker and less automatic compared with that of facial expression processing (Frühholz et al., 2011). The ENP effect of electroencephalography indicators was documented to appear only in response to emotional faces but not to emotional words (Rellecke et al., 2011).

Moreover, in the processing of emotional words, positive words induced a larger amplitude than negative words and neutral words (Scott et al., 2009); in contrast, in the processing of emotional faces, negative expressions induced a larger amplitude than positive and neutral expressions, showing a negative emotion processing advantage (Bayer & Schacht, 2014). In conclusion, emotional facial expressions can be automatically processed and possess the advantage of negative emotional processing. Emotional faces convey more reliable emotional information than emotional words and have higher emotional arousal (Levens & Gotlib, 2010; Wang, Lu, et al., 2019; Yuan et al., 2019).

Based on the contradiction between visual attention deficit theory and visual attention compensation theory, the current study used emotional image library to analyze fearful and disgustful facial expressions, and combined with the RSVP paradigm to explain the differences in emotional attentional blink between deaf and hearing children. In the meantime, the applicability between visual attention deficit theory and visual attention compensation theory can also be tested.

## Method

### Participants

G*Power3.1 was used to calculate the sample size required for the study (Faul et al., 2007). The following parameters were used: effect size = 0.2, α = .05, 1 − β = 0.85 (Zhang & Zuber, 2020); the number of groups was 2, and the total sample size was 40. Finally, 43 eligible participants were recruited, including 23 deaf children (11 female, 12 male) and 20 hearing children (11 female, 9 male). Deaf children (congenital deaf and signers) were selected according to the classification standard of hearing loss of the World Health Organization (WHO) in 2021, with hearing loss above 95 dB. The average age of the deaf children and the hearing group was 14.10 (*SD *= 0.64) and 14.26 (*SD *= 0.62). All participants were healthy without genetic diseases, with normal vision, or corrected vision, right-handed, and had not participated in similar experiments.

### Design and Materials

This study adopted a mixed experimental design: Participant Type 2 (deaf children, hearing children) × T1 Facial Emotion Type 2 (fear, disgust) × T2 Delay Position 2 (Lag2, Lag6). Among them, T1 facial expression type and T2 delay position were within-subject variables, and subject type was between-subject variable. The dependent variable is the response accuracy of T1 and T2 (T2 accuracy was calculated for T1-correct trials only).

Fear, disgust, and neutral pictures were selected from the picture system image set by Gong et al. (2011). In the training phase, two faces with fearful and disgustful expressions were selected for T1, two neutral faces were selected for T2, and 16 neutral faces were selected for distraction stimuli. In the formal experiment, 10 fearful and disgustful faces were selected for T1, 10 faces with fearful and disgustful expressions were selected for T2, and 16 neutral faces were selected for the distraction stimuli. Another 30 participants (14 deaf children, 16 hearing children), including 15 females (seven deaf children; *M *= 14.33 *SD *= 0.62) and 15 males (seven deaf children; *M *= 14.20 *SD *= 0.68), were asked to rate the valence, arousal, and facial attractiveness of fear, disgust, and neutral pictures by self-reporting on a 9-point scale. The results of variance analysis showed that the valence scores of the three types of pictures were significantly different, *F*(2, 1,437) = 987.61, *p *< .001, *η2 p *= 0.58. Multiple comparisons showed that there was no significant difference in the valence scores of faces with fearful and disgustful expressions (*p *= .295); however, they were higher than for neutral faces (*p_S _*< .001). There was a significant difference in the arousal scores of the three types of pictures, *F*(2, 1,437) = 1,703.09, *p *< .001, *η2 p *= 0.70. Multiple comparisons showed that there was no significant difference in arousal scores between faces with fearful and disgustful expressions (*p *= .45). However, both of them were higher than for neutral faces (*p_S _*< .001). There was no significant difference in facial attractiveness among the three types of pictures (*p *= .457).

### Procedure

**Figure 1.**
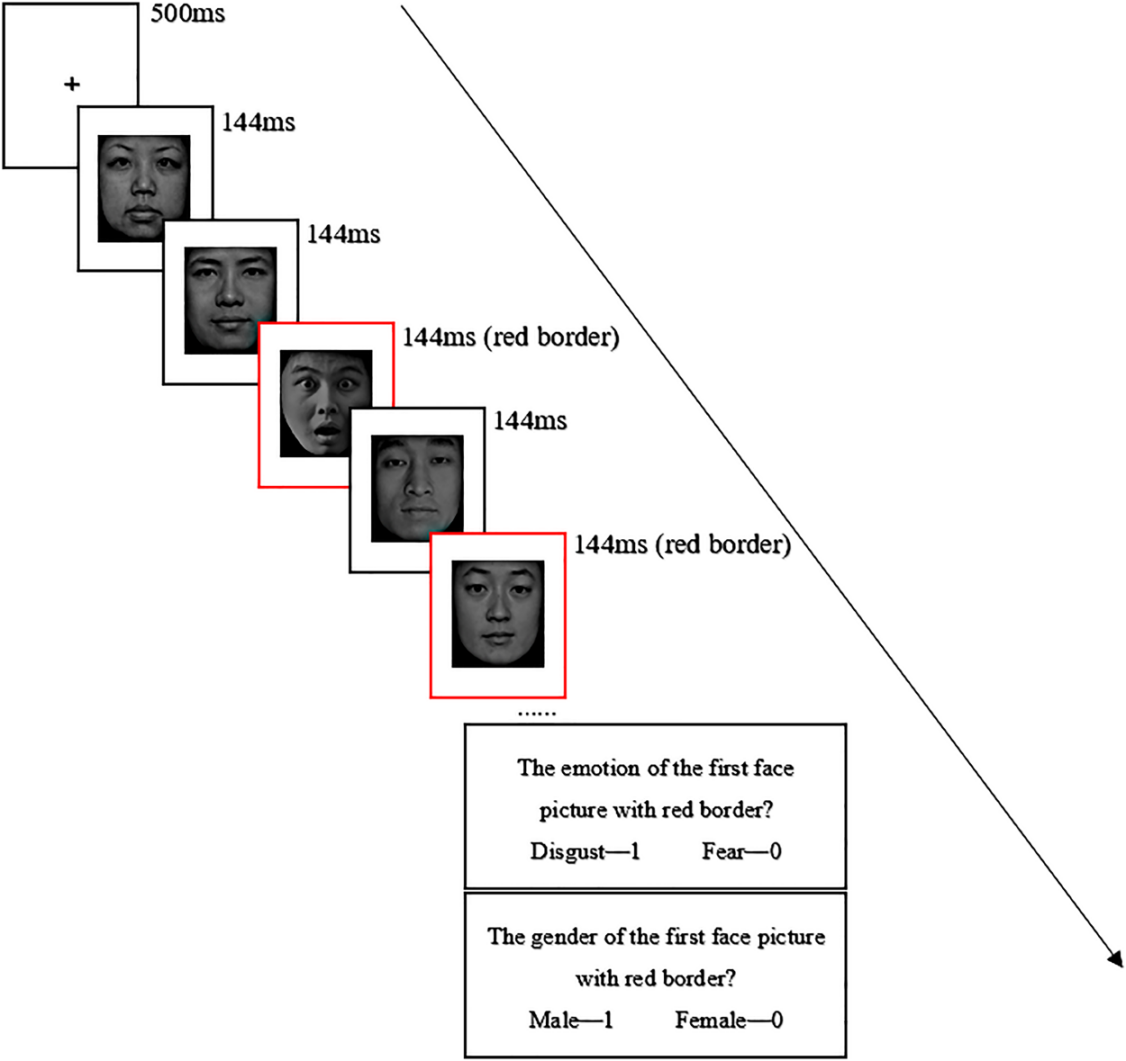
The trial procedure for the attentional-blink paradigm.

The stimuli were presented in the center of 16-in. computers. The distance between the participants and the computer screen was about 80 cm. The device resolution was 1,024 × 768, and the refresh rate was 60 Hz. The experimental program is designed and presented using E-Prime 3.0. First, a fixation point of “+” was presented on the screen for 500 ms, followed by the RSVP stimulus stream composed of facial expression images (the gender ratio of the stimuli is equal), in which T1 and T2 borders were red, and each image presented at 144 ms. T1 was presented at the third or sixth position in the RSVP series; T2 lagged T1 by appearing at the second (Lag2) or the sixth position (Lag6). The training phase included 10 repeatable trials, and the formal experiment included 72 trials in total. After each trial, the subjects were asked to judge the emotion type of T1 by pressing 1 for facial expression of disgust and 0 for facial expression of fear. To determine the gender of T2, subjects were asked to press 1 for male and 0 for female. The participants were tested separately in a quiet and well-lit laboratory, 60 cm away from the screen, and the experiment lasted about 15 min. The time setting of image presentation in this experiment referred to the study of Trippe et al. (2007). The experimental process is shown in Figure 1.

**Figure 2.**
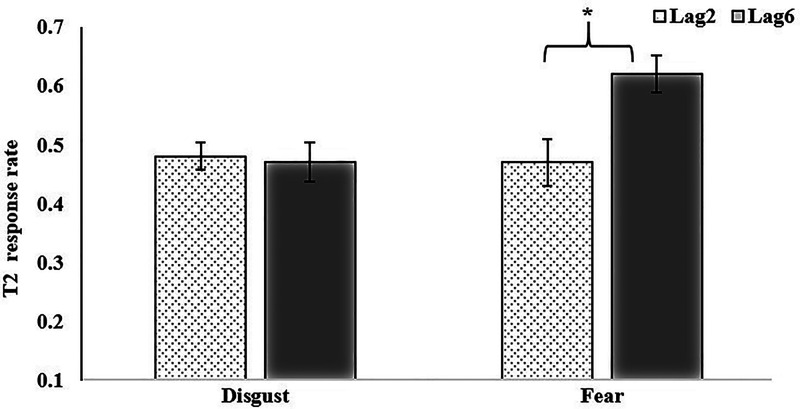
The interaction between T1 facial emotion type and T2 position Note: *p＜.05.

**Table 1.** Response accuracy of T1 and T2 in different lags in deaf and hearing children (*M ± SD*).

Participant type	Emotion of T1	T1	lag2	lag6
Deaf children	Fear	0.49 ± 0.22	0.46 ± 0.24	0.64 ± 0.22
	Disgust	0.58 ± 0.23	0.46 ± 0.14	0.48 ± 0.26
Hearing children	Fear	0.51 ± 0.18	0.48 ± 0.19	0.61 ± 0.19
	Disgust	0.63 ± 0.17	0.49 ± 0.16	0.46 ± 0.27

## Results

Levene's test for homogeneity of variance showed homogeneity of variance(*p = *.619). Repeated-measures ANOVA for T1 showed that the main effect of “facial emotion type” was significant, *F*(1, 41) = 3.28, *p *= .08, *η2 p *= 0.07, *BF_10 _*= 2.67. The accuracy rate for T1 with a disgustful face (*M *= 0.60, *SD *= 0.23) was higher than T1 with a fearful face (*M *= 0.50, *SD *= 0.17). The main effect of participant type was not significant, *F*(1, 41) = 0.20, *p *= .16, *η2 p* = 0.05. The interaction between facial emotion type and participant type was not significant. *F*(1, 41) = 0.08, *p *= .78, *η2 p* ＜ 0.01.

Repeated-measures ANOVA for T2 showed that the main effect of “facial emotion type” on T1 was significant, *F*(1, 41) = 8.15, *p *< .01, *η2 p *= 0.17, *BF_10 _*= 1.51. When T1 presents a fearful face, the accuracy rate for T2 (*M *= 0.55, *SD *= 0.20) was higher than T2 when T1 presents a disgustful face (*M *= 0.47, *SD *= 0.21). The main effect of “T2 position” was significant, *F*(1, 41) = 5.84, *p *< .05, *η2 p *= 0.13, *BF_10 _*= 1.63. When the T2 position was at lag6, the response accuracy (*M *= 0.55, *SD *= 0.23) was higher than at Lag2 (*M *= 0.47, *SD *= 0.18). The interaction between facial emotion type at T1 and T2 positions was significant. *F*(1, 41) = 4.63, *p *< .05, *η2 p *= 0.10, *BF_10 _*= 8.20. Simple effect analysis showed that when T2 appeared at Lag6, the response accuracy of T2 in disgust T1 condition was lower than that in the fear T1 condition. However, no significant difference for T2 at Lag2 was found between the two conditions (see Figure 2; Table 1).

The main effect of participant type was not significant, *F*(1, 41) ＜ 0.01, *p *= .97, *η2 p* ＜ 0.01. The interaction between facial emotion type at T1 and participant type was significant. *F*(1, 41) = 0.01, *p *= .91, *η2 p* ＜ 0.01. The interaction between the T2 position and participants type was not significant, *F*(1, 41) = 0.53, *p *= .47, *η2 p *= 0.01. The three-way interaction of participants’ type and facial emotion type at T1 and T2 position was not significant, *F*(1, 41) ＜ 0.01, *p *= .94, *η2 p* ＜ 0.01.

## Discussion

Herein, the RSVP paradigm was used to compare the differences in an attentional blink for facial expressions of disgust and fear between deaf and hearing children aged 13 to 15 years. The results showed better recognition of disgustful faces in deaf and hearing children. When T2 appeared at Lag6, the response accuracy of T2 in disgust T1 condition was lower than that in fear T1 condition, however, no significant difference for T2 at Lag2 was found between the two conditions. There was no significant difference in attentional blink between deaf and hearing children.

Firstly, the present study found that deaf and hearing children had a higher accuracy rate of disgustful face than fearful face at T1, consistent with the literature. Relevant studies found that under the condition of sufficient attention resources, participants’ recognition of disgust was better than that of fear (Chapman et al., 2013; Ding et al., 2018). At the same time, our study also corroborated that the attentional processing time of disgustful emotion was longer, and it was more difficult to disengage attention, which led to a decrease in the response accuracy of T2. Carretié et al. (2011) used the cost-benefit principle to explain this phenomenon, arguing that the difference between fear and disgust arises from calculating gains and losses and that careful observation of resources invested in disgustful situations may yield gains. For example, observing some disgusting foods can be avoided. To reduce the loss, the allocation of attention is often decreased. However, some studies have found that fear interferes more with subsequent tasks than disgust (Finucane, 2011). Conversely, Van Hooff et al. (2014) pointed out that only when the stimulus arouses enough fear, even to the point of inducing life-threatening fear, will the phenomenon of fear interference effect be more obvious. Whereas, to avoid the psychological discomfort caused by the experiment, the present study did not select fearful pictures with a sense of life threat. Further studies should be carried out to explore the fear of stimulus in detail.

Moreover, as mentioned above, there are two different views on the visual attention ability of deaf children, namely, attention deficit theory and attention compensation theory (Daza & Phillips-Silver, 2013). In this study, deaf children were not found to have more attentional blinks than hearing children, which indicated that deaf children did not have a certain deficit in visual attention to facial expressions, which is consistent with findings (aged 18–40) of a study by Dye and Bavelier (2010). In contrast, from the perspective of integration, some studies have pointed out that the reason for the divergence between attention deficit and compensation theory may be that the experimental materials and procedures used in each study are not completely the same (Thakur et al., 2019). From the perspective of cognitive control, the inability of participants to efficiently eliminate the interference of distracting stimuli causes the attentional blink (Olivers & Meeter, 2008; Olivers & Nieuwenhuis, 2006). Previous studies have also confirmed that distracting stimuli play a key role in attentional blink (Maki et al., 2003; Xia et al., 2022). The attentional blink paradigm used in this study is relatively complex, and the stimulus presentation time is short. Therefore, the average accuracy of some experimental results was lower than 50%, indicating that the experimental difficulty was higher both for deaf and hearing children. Moreover, previous studies have confirmed that attentional blink is more likely to occur under difficult task conditions (Christmann & Leuthold, 2004; Shi et al., 2020; Zhang & Wang, 2009). Finally, hearing children in the adolescent stage are also prone to negative emotions (Díaz-Geada et al., 2019; Zhang et al., 2022). Adolescents are in the peak period of brain development, and the relevant studies found that the auditory cortex of deaf adolescents processed cross-channel reorganization (Merabet & Pascual-Leone, 2010). As a result, deaf adolescents could process visual information through the auditory cortex (Almeida et al., 2015; Bottari et al., 2014). Therefore, excessive cognitive resources are invested in T1 negative facial emotion and the development of the brain, which may explain the lack of significant difference in attentional blink between deaf children and hearing children.

Previous studies have used emotional words as experimental materials to explore the phenomenon of emotional attentional blink in deaf children (Wang, Wang, et al., 2019). This study expands the research field of attentional blink in deaf children by using facial expressions. Facial expression stimulation is closer to daily life, and the emotional information conveyed is more explicit. Compared with emotional words, the emotional effect generated by facial expression is more intense (Frühholz et al., 2011). Moreover, facial emotion possesses automatic processing characteristics (Stenberg et al., 1998) and the advantage of negative emotion processing (Bayer & Schacht, 2014).

Body expression refers to the emotions and a series of coordinated and meaningful actions expressed by the body (De Gelder, 2006). Intriguingly, the body conveys more explicit information than facial expressions, and individuals can judge their emotional types at a longer distance (van de Riet et al., 2009). Meanwhile, the body can activate the precuneus, fusiform gyrus, striatal body area, temporal, parietal lobe, superior parietal lobule, primary somatosensory cortex, and thalamus more significantly than facial expression (Kret et al., 2011), and also induce the P1 component of occipital lobe more significantly (Fang et al., 2019). Based on this, future research should compare the attentional blink for body expression between deaf and hearing children. Additionally, different types of emotional materials could be used to explore the brain mechanism of visual attention processing in deaf children. In the meantime, subsequent multi-baseline or longitudinal intervention studies can be carried out around “facial expression attention training" (De Voogd et al., 2017; LeMoult et al., 2016).

Last but not least, the stimulus in previous studies of deaf children’s attentional blink was presented at the center of the visual field (e.g., Jia et al., 2016; Raymond et al., 1992; Wang et al., 2019). Some other studies also found that deaf individuals redistribute attentional resources from the center of the visual field to the periphery (Bavelier et al., 2000; Codina et al., 2011; Dye & Hauser, 2014). However, this difference was not the focus of the current study. Aiming at the problem of the visual field, we expect a new paradigm can be produced to exclude this extra variable from the experimental results.

### Conclusion

Our results found that deaf and hearing children were more sensitive to facial expressions of disgust, which captured more attentional resources, and the ability of visual attention of deaf children was not weaker than hearing children.
